# Supplementary material for: Accelerating water dissociation kinetics by isolating cobalt atoms into ruthenium lattice
Source: Nat Commun. 2018 Nov 23;9:4958. doi: 10.1038/s41467-018-07288-6 (PMC6251903; doi:10.1038/s41467-018-07288-6)
Supplement: Supplementary file 1 — Supplementary Information [file 41467_2018_7288_MOESM1_ESM.pdf]

Supplementary Information

Accelerating Water Dissociation Kinetics by Isolating Cobalt Atoms into Ruthenium Lattice

Mao et al

Supplementary Figures and Tables

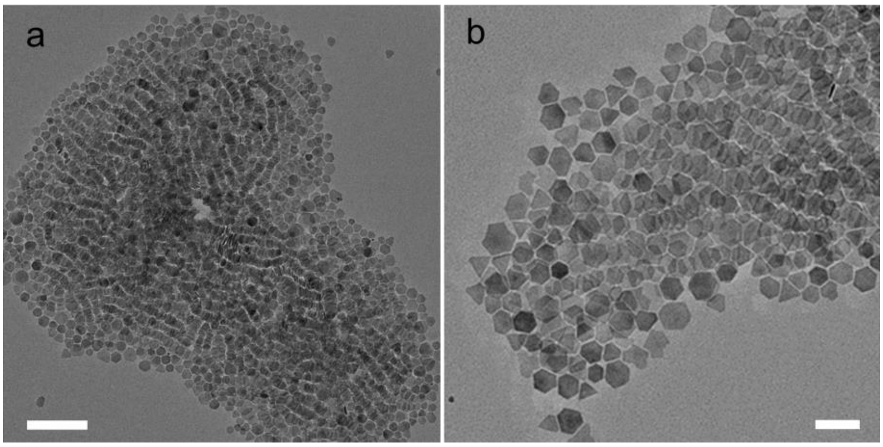

Supplementary Figure 1. TEM images of Co-substituted Ru nanosheets with different magnifications. **a** Scar bar: 200 nm. **b** Scar bar: 60 nm.

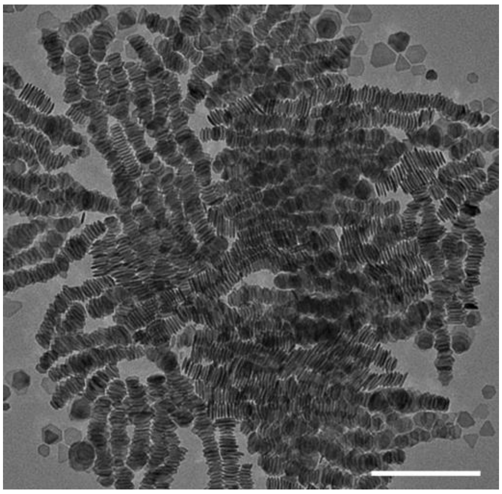

Supplementary Figure 2. TEM image of the assembly of Co-substituted Ru nanosheets, scar bar: 200 nm.

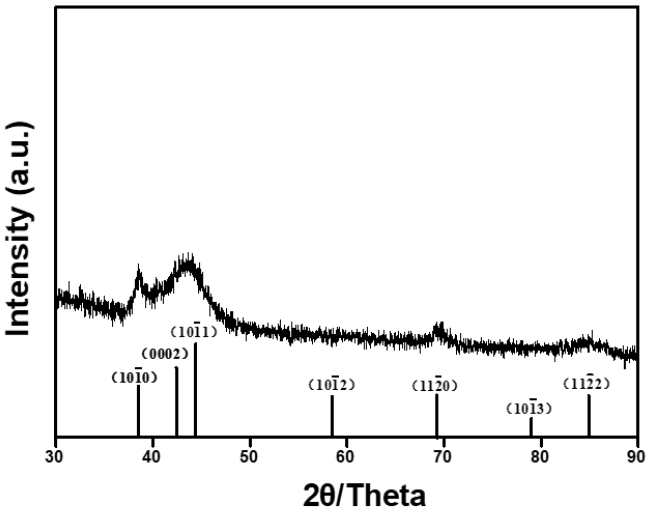

Supplementary Figure 3. XRD pattern of Co-substituted Ru nanosheets. The standard pattern of Ru (JCPDS card No. 06-0663) are shown beneath the plot.

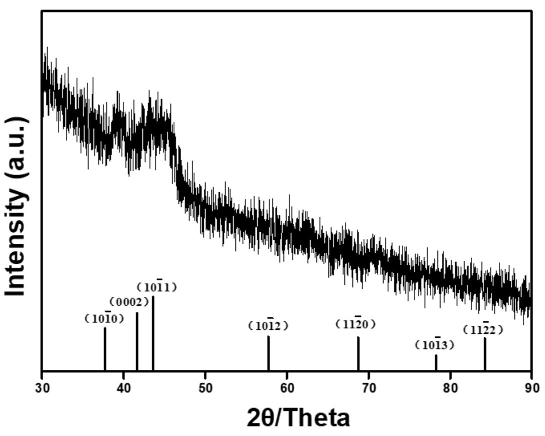

Supplementary Figure 4. XRD pattern of RuCo alloy. The standard pattern of Ru (JCPDS card No. 06-0663) are shown beneath the plot.

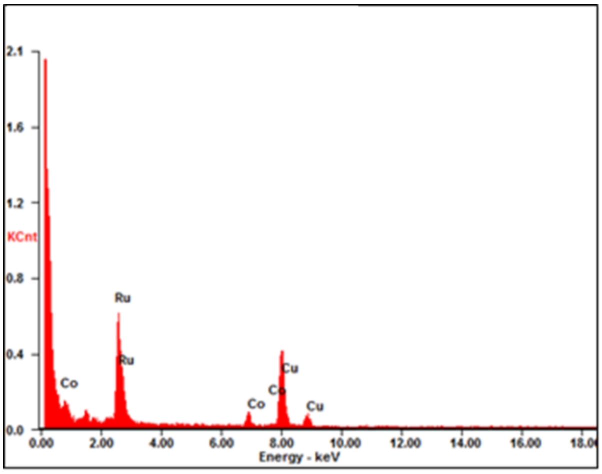

Supplementary Figure 5. EDX pattern of Co-substituted Ru nanosheets. The Ru: Co atomic ratio was 94 : 6.

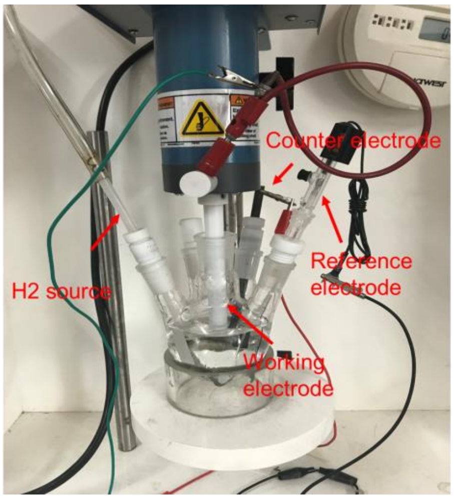

Supplementary Figure 6. Photograph of the three-electrode system for the electrochemical test.

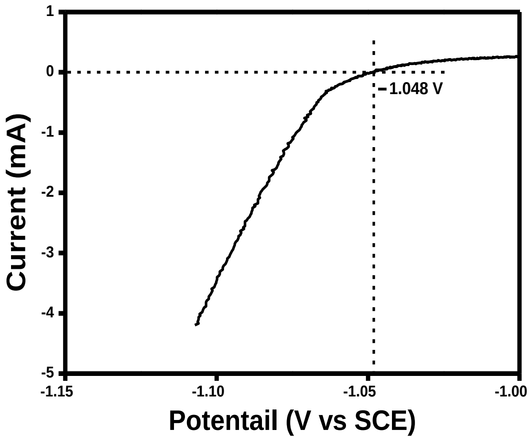

Supplementary Figure 7. LSV curves of Pt in 1.0 M KOH solution (H_2-saturated), used for calibration of the SCE with respect to RHE. Scan rate: 5 mV s^{-1}.

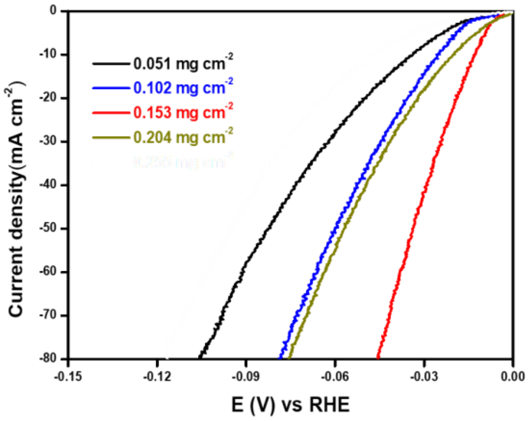

Supplementary Figure 8. LSV curves of Co-substituted Ru catalyst at different loading weight in 1.0 M KOH. Scan rate: 5 mV s^{-1}.

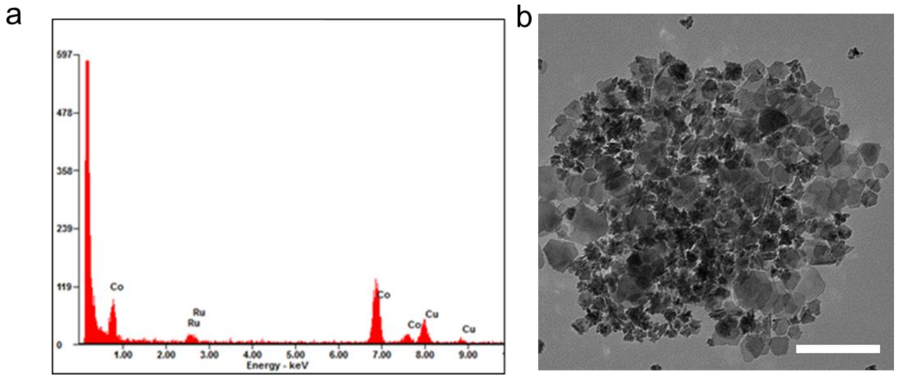

Supplementary Figure 9. a EDX analysis. Ru : Co atomic ratio was 32:68 (close to 1:2). **b** TEM image of $RuCo_2$ catalyst. Scar bar:100 nm.

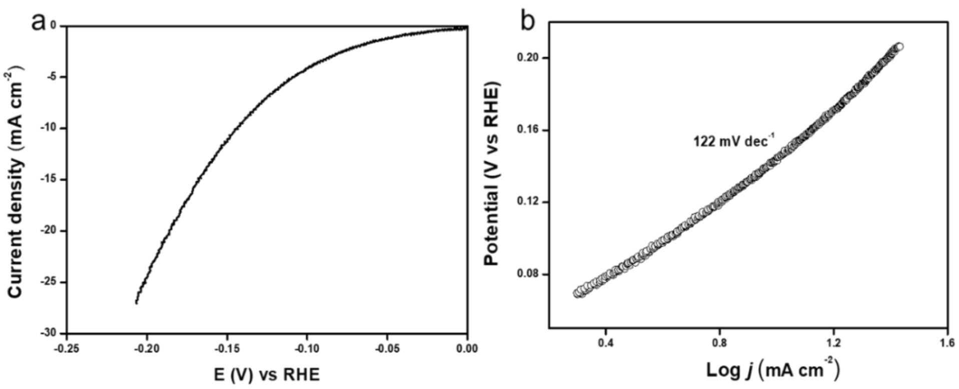

Supplementary Figure 10. a LSV curves of $RuCo_2$ catalyst in 1 M KOH solution. **b** Tafel plots of the polarization curves in a.

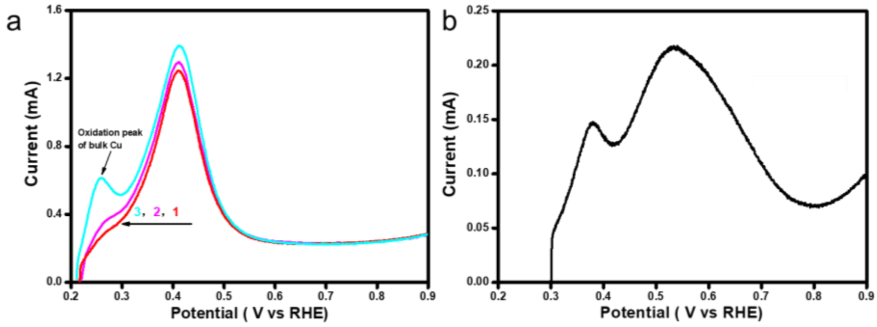

Supplementary Figure 11. Copper UPD in the presence of 5 mM $CuSO_4$ on **a** Co-substituted Ru, and **b** Pt/C catalyst.

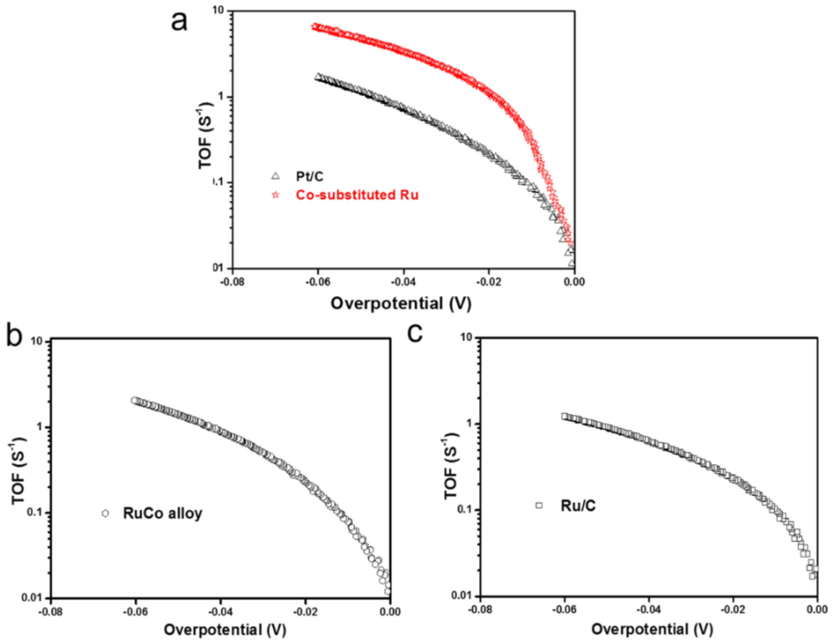

Supplementary Figure 12. TOF values of **a** Co-substituted Ru (red line), Pt/C (black line), **b** RuCo alloy and **c** Ru/C catalyst.

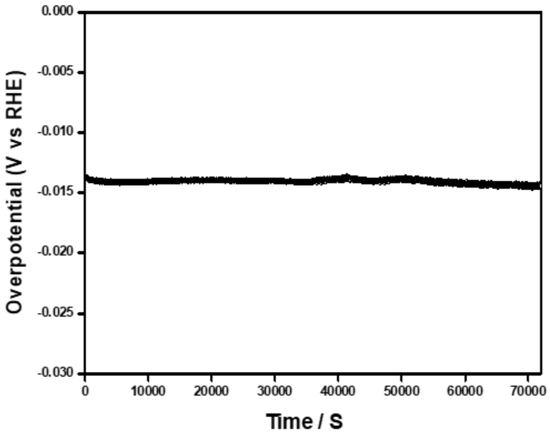

Supplementary Figure 13. Chronopotentiometric curves of Co-substituted Ru catalyst recorded at a constant current density of 10 mA cm^{-2} in 1.0 M KOH.

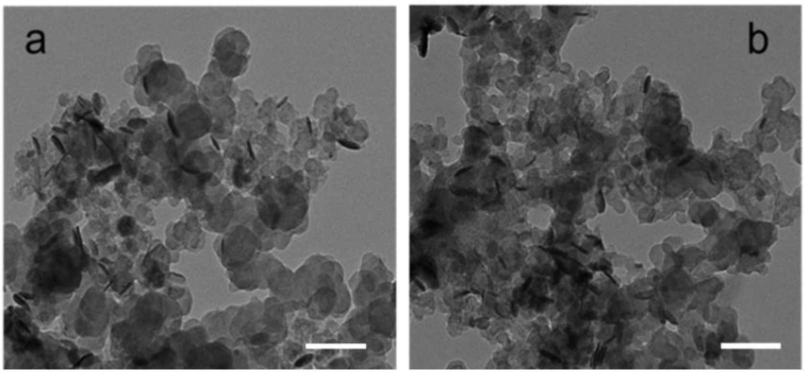

Supplementary Figure 14. TEM images of the Co-substituted Ru /C catalyst **a** before and **b** after electrochemical test. Scar bar: 100 nm.

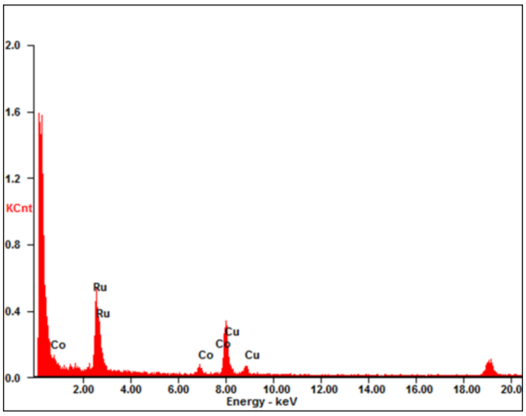

Supplementary Figure 15. EDS analysis of Co-substituted Ru catalyst after electrochemical test. The Ru : Co atomic ratio was 94 : 6.

Supplementary Table 1.

Comparison of HER performance between Co-substituted Ru NSs and other reported Pt-free catalysts in alkaline medium.

Catalysts	Electrode	Loading amount (mg cm^{-2})	Electrolyte	Overpotential at 10 $mAcm^{-2}$ (mV)	Tafel plots ($mVdec^{-1}$)	Ref.
Co-substituted Ru	GC	0.153	1 M KOH	13	29	This work
CoOx@CN	GC	0.42	1 M KOH	232	115	1
Co/Co_3O_4	NF	0.85	1 M KOH	90	44	2
$Ru/C_3N_4/C$	GC	0.204	0.1 M KOH	79	/	3
MoP@RGO	GC	0.28	1 M KOH	93 (η_{20})	58	4
$Zn_{0.3}Co_{2.7}S_4$	GC	0.285	1 M KOH	85	/	5
Co_2P@NPG	GC	0.5	1 M KOH	165	96	6
$Mo_1N_1C_2$	GC	0.408	0.1 M KOH	132	90	7
$FePO_4$/NF	NF	0.285	1 M KOH	123	104.49	8
$FeSe_2$	NF/FTO	3/1	1 M KOH	178/235	/	9
CoP NWs 40s/CC	CC	N/A	1 M KOH	146 (η_{100})	42.8	10
Mo_2N-Mo_2C/HGr-3	GC	0.337	1 M KOH	154	68	11
MoB/g-C_3N_4	GC	0.25	1 M KOH	133	46	12
RuP_2 @NPC	GC	1.0	1 M KOH	52	69	13
RuCo@NC	GC	0.275	1 M KOH	28	31	14
Ru@C_2N	GC	0.285	1 M KOH	17	38	15
CoP/rGO-400	GC	0.28	1 M KOH	150	38	16
MoCx nano octahedrons	GC	0.8	1 M KOH	151	59	17
IrCo@NC-500	GC	0.285	1 M KOH	45	80	18
RuCoP	CNF	0.3	1 M KOH	23 mV	37	19

Note: GC stands for glassy carbon. NF stands for nickel foam; FTO stands for fluorine-doped tin oxide. CC stands for carbon cloth. CNF stands for carbon nanofiber. "/": the data was not given.

Supplementary References

1. Jin, H. et.al. In situ Cobalt-Cobalt Oxide/N-Doped Carbon Hybrids As Superior Bifunctional Electrocatalysts for Hydrogen and Oxygen Evolution. *J. Am. Chem. Soc.* **137,** 2688-2694 (2015).
2. Yan, X., Tian, L., He, M. & Chen, X. Three-Dimensional Crystalline/Amorphous Co/Co3O4 Core/Shell Nanosheets as Efficient Electrocatalysts for the Hydrogen Evolution Reaction. *Nano Lett.* **15,** 6015-6021 (2015).
3. Zheng, Y. et.al. High Electrocatalytic Hydrogen Evolution Activity of an Anomalous Ruthenium Catalyst. *J. Am. Chem. Soc.* **138,** 16174-16181 (2016).
4. Zhang, G. et.al. Highly Active and Stable Catalysts of Phytic Acid-Derivative Transition Metal Phosphides for Full Water Splitting. *J. Am. Chem. Soc.* **138,** 14686-14693 (2016).
5. Huang, Z. et.al. Hollow Cobalt-Based Bimetallic Sulfide Polyhedra for Efficient All-pHValue Electrochemical and Photocatalytic Hydrogen Evolution. *J. Am. Chem. Soc.* **138,** 1359-1365 (2016).
6. Zhuang, M. et.al. Polymer-Embedded Fabrication of Co_2P Nanoparticles Encapsulated in N,P-Doped Graphene for Hydrogen Generation. *Nano Lett.* **16,** 4691-4698 (2016).
7. Chen, W. et al. Rational Design of Single Molybdenum Atoms Anchored on N-Doped Carbon for Effective Hydrogen Evolution Reaction. *Angew. Chem. Int. Ed.* **129,** 16302-16306 (2017).
8. Yang, L. et al. Vertical Growth of 2D Amorphous $FePO_4$ Nanosheet on Ni Foam: Outer and Inner Structural Design for Superior Water Splitting. *Adv. Mater.* **29,** 1704574-1704582 (2017).
9. Panda, C. et al. From a Molecular 2Fe-2Se Precursor to a Highly Efficient Iron Diselenide Electrocatalyst for Overall Water Splitting. *Angew. Chem. Int. Ed.* **56,** 10506-10510 (2017).
10. Xu, K. et al. Controllable Surface Reorganization Engineering on Cobalt Phosphide Nanowire Arrays for Efficient Alkaline Hydrogen Evolution Reaction *Adv. Mater.* **30,** 1703322-1703327 (2017).
11. Yan, H. Holey Reduced Graphene Oxide Coupled with an Mo_2N-Mo_2C Heterojunction for Efficient Hydrogen Evolution *Adv. Mater.* **30,** 1704156-1704163 (2018).
12. Zhuang, Z. et al. MoB/g-C_3N_4 Interface Materials as a Schottky Catalyst to Boost Hydrogen Evolution. *Angew. Chem. Int. Ed.* **57,** 496-500 (2018).
13. Pu, Z. et al. RuP_2-Based Catalysts with Platinum-like Activity and Higher Durability for the Hydrogen Evolution Reaction at All pH Values *Angew. Chem. Int. Ed.* **56,** 11559-11564 (2017).
14. Su, J. et al. Ruthenium-cobalt nanoalloys encapsulated in nitrogen-doped graphene as active electrocatalysts for producing hydrogen in alkaline media. *Nat. Commun.* **8,** 14969-14978 (2017).
15. Mahmood, J. et al. An efficient and pH-universal ruthenium-based catalyst for the hydrogen evolution reaction. *Nat. Nanotechnol.* **12,** 441-446 (2017).
16. Jiao, L., Zhou, Y. & Jiang, H. Metal-organic framework-based CoP/reduced graphene oxide: high-performance bifunctional electrocatalyst for overall water splitting. *Chem. Sci.,* **7,** 1690-1695 (2016).
17. Wu, H. et al. Porous molybdenum carbide nano-octahedrons synthesized via confined carburization in metal-organic frameworks for efficient hydrogen production. *Nat. Commun.*

6, 6512-6519 (2015).
18. Jiang, P. et al. Tuning the Activity of Carbon for Electrocatalytic Hydrogen Evolution via an Iridium-Cobalt Alloy Core Encapsulated in Nitrogen-Doped Carbon Cages. *Adv. Mater.* **30,** 1705324-1705334 (2018).
19. Xu, J. et al. Boosting the hydrogen evolution performance of ruthenium clusters through synergistic coupling with cobalt phosphide. *Energy Environ. Sci.* **11,** 1819-1827 (2018).
